# Incidental findings and safety events from magnetic resonance imaging simulation for head and neck radiation treatment planning: A single institution experience

**DOI:** 10.1016/j.tipsro.2023.100228

**Published:** 2023-12-12

**Authors:** Jonathan Massachi, Lisa Singer, Christine Glastonbury, Jessica Scholey, Kamal Singhrao, Christina Calvin, Sue S. Yom, Jason W. Chan

**Affiliations:** a Department of Radiation Oncology, University of California, San Francisco, San Francisco, CA, USA; b Department of Radiology, University of California, San Francisco, San Francisco, CA, USA

**Keywords:** MRI, MRI-Sim, Treatment Planning, Head and Neck Tumors, Incidental Findings, Safety, Radiation Therapy

## Abstract

**Purpose:**

Having dedicated MRI scanners within radiation oncology departments may present unexpected challenges since radiation oncologists and radiation therapists are generally not trained in this modality and there are potential patient safety concerns. This study retrospectively reviews the incidental findings and safety events that were observed at a single institution during introduction of MRI sim for head and neck radiotherapy planning.

**Methods:**

Consecutive patients from March 1, 2020, to May 31, 2022, who were scheduled for MRI sim after having completed CT simulation for head and neck radiotherapy were included for analysis. Patients first underwent a CT simulation with a thermoplastic mask and in most cases with an intraoral stent. The same setup was then reproduced in the MRI simulator. Safety events were instances where scheduled MRI sims were not completed due to the MRI technologist identifying MRI-incompatible devices or objects at the time of sim. Incidental findings were identified during weekly quality assurance rounds as a joint enterprise of head and neck radiation oncology and neuroradiology. Categorical variables between completed and not completed MRI sims were compared using the Chi-Square test and continuous variables were compared using the Mann-Whitney *U* test with a p-value of < 0.05 considered to be statistically significant.

**Results:**

148 of 169 MRI sims (88 %) were completed as scheduled and 21 (12 %) were not completed (Table 1). Among the 21 aborted MRI sims, the most common reason was due to safety events flagged by the MRI technologist (n = 8, 38 %) because of the presence of metal or a medical device that was not noted at the time of initial screening by the administrative coordinator. Patients who did not complete MRI sim were more likely to be treated for non-squamous head and neck primary tumor (p = 0.016) and were being treated post-operatively (p < 0.001). CT and MRI sim scans each had 17 incidental findings. CT simulation detected 3 cases of new metastases in lungs, which were outside the scan parameters of MRI sim. MRI sim detected one case of dural venous thrombosis and one case of cervical spine epidural abscess, which were not detected by CT simulation.

**Conclusions:**

Radiation oncology departments with dedicated MRI simulation scanners would benefit from diagnostic radiology review for incidental findings and having therapists with MRI safety credentialing to catch near-miss events.

## Highlights

• There is significant interest in replacing CT simulation with MR-only simulation for head and neck radiotherapy.
• Since radiation oncologists are generally more familiar with CT than MRI, incidental findings from MRI sim may be more likely to be unrecognized without diagnostic radiology support.
• Incidental findings were observed in similar proportions from CT or MRI sim (11%).
• MRI sim detected non-oncologic important incidental findings in two patients that were not observed on the corresponding CT sim, both of which were identified by a neuroradiologist at the time of contour review.

## Introduction

There has been rapid growth in the use of magnetic resonance imaging (MRI) for head and neck radiotherapy planning and treatment delivery [1]. Compared to computed tomography (CT), MRI offers improved soft tissue contrast resolution which allows more accurate determination of the extent of the primary tumor and /or perineural tumor extension. Furthermore, MRI can also be less prone to artifacts from dental amalgam, which facilitates more accurate organs at risk (OAR) and target volume delineation [2], [3], [4].

Traditionally, CT simulation has been considered essential as Hounsfield Units from CT images are converted to electron densities used for dose calculation. As such, the incorporation of MRI in head and neck radiotherapy planning has generally been limited to fusion of diagnostic MRIs to CT simulation images to aid in contouring. However, errors can arise from differences in patient positioning when the MRI and CT studies are obtained [5], [6], [7]. MR-only radiotherapy workflows has therefore been proposed to avoid the geometric uncertainties of combining MRI with CT [8].

In recent years, there has been rapid technological development of MRI-guided radiotherapy (MRgRT) and MRI simulations (MR sim) that are paving the way for MR-only radiotherapy. MRgRT delivered with linear accelerators with on-board MRI scanners (MR-Linac) has demonstrated early promising indications of safety and effectiveness in many tumor types [9], [10]. Furthermore, synthetic CT (sCT) generation from MR sim images using deep learning and convoluational neural networks can be used for electron density estimation and dose calculation in treatment planning. sCT generation is an active area of research and several different methods have demonstrated feasibility in using MRI sim only for accurate absorbed dose calculations for head and neck radiotherapy planning [11], [12], [13].

As more radiation oncology departments develop and adopt MR-only workflows, radiotherapy professionals will be confronted with new challenges posed by MRI technologies. Having dedicated MRI scanners within radiation oncology departments may present unexpected challenges since radiation oncologists and radiation therapists are generally not trained in this modality and there are potential patient safety concerns [14]. In this study, we report the incidental findings and safety events that were observed in our initial experience with MRI sim for head and neck radiotherapy.

## Methods

### MRI simulation workflow

**Fig. 1:**
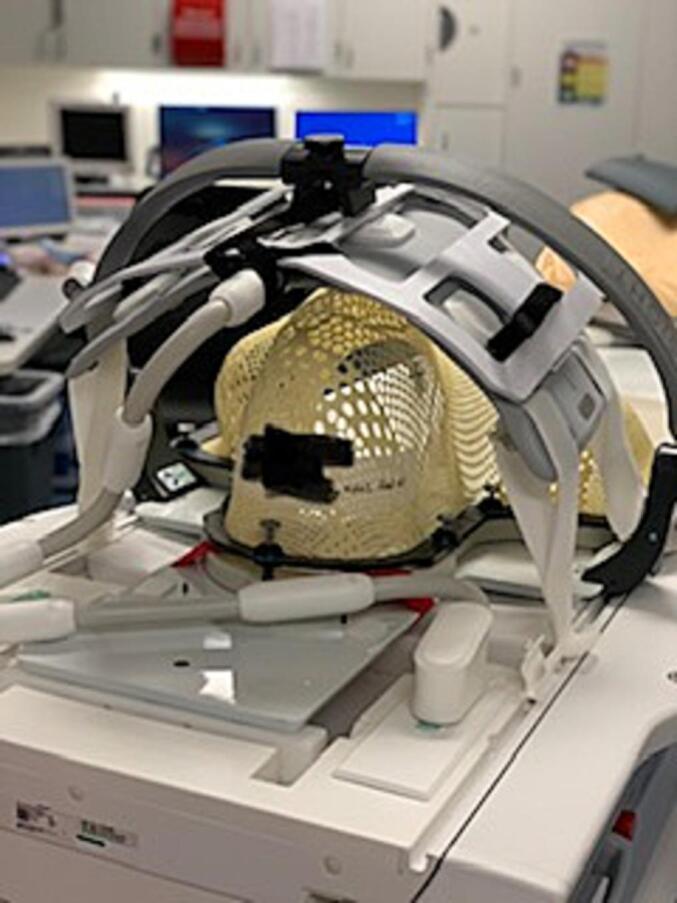
MRI simulation setup. In order to accommodate the thermoplastic mask and intraoral stent, two UltraFlex Large 18 coils were used with torso and spine coils.

Our radiation oncology department installed a 3 Tesla MRI simulator (MAGNETOM Vida, Siemens Healthcare, Erlangen, Germany) in late 2019 and started clinical MRI sims in early 2020. Patients first underwent a CT simulation with a thermoplastic mask and, in most cases, with an intraoral stent. The same setup was then reproduced in the MRI simulator (Fig. 1). In most cases, CT simulations imaged from vertex scalp to lungs while MRI sims imaged from orbits to clavicles. In order to accommodate the thermoplastic mask and intraoral stents, two UltraFlex Large 18 coils were used with torso and spine coils. Localizer, T2 fat-saturated, diffusion-weighted, T1 volumetric interpolated breath hold examination (VIBE) pre-contrast, with or without T1 VIBE Dixon post-contrast sequences were taken. Rigid registrations were performed to fuse CT simulation images to T2, T1 pre-, and T1 post-contrast simulation images for contouring.

### MRI safety screening

A standard hospital-approved MRI screening form to identify patients with metallic devices that are not MRI compatible was completed with patients by administrative coordinators at the time of scheduling CT and MRI sim studies. The screening form was reviewed by the MRI technologist prior to allowing patients to enter Zone 3 and 4 in accordance with safety guidelines by the American College of Radiology [15]. The MRI technologist was also certified as a Magnetic Resonance Safety Officer [16]. Patients with claustrophobia were offered an anxiolytic prior to CT and MRI sim studies. Our institution’s guidelines for screening, training, and staffing of MRI sim for head and neck radiotherapy are listed in Table 1.

**Table 1:** Screening, Training, and Staffing of MRI Sim for Head and Neck Radiotherapy.

Patient Screening	• A standard hospital approved MRI screening form is filled out by patient or by healthcare provider prior to patient entering Zone 3/4. Implants require review of manufacturing label before scheduling. MRI technologist reviews the MRI screening form prior to allowing patient to enter Zone 3/4.
If Unable to Complete MRI Screening	• A knowledgeable family member, caretaker, or healthcare provider can complete the screening form on behalf of a patient. In the absence of sufficient history, the following steps are required: Attending radiologist approval for MRI along with documentation of medical necessity by attending physician requesting the study. Plain film x-rays prior to MRI to ensure absence of contraindicated devices or other contraindicated metallic materials. Screening head or orbit CT prior to MRI for metallic foreign bodies. The scout image for this CT can be used to screen for aneurysm clips.
MRI Personnel Screening	• A hospital-approved screening form will be kept on file for every MRI personnel. All MRI personnel must report to their supervisor any trauma, procedure, or surgery that they experience with a ferromagnetic metallic object/device that may have been introduced within or on them.
Training	• Quarterly meetings between MRI safety officer (MRSO) and staff that work in MR arena. Monthly meetings between MRSO and radiation oncology clinical and physics leads in MRI. All new hires including nurses, patient navigators, and MRI technologists undergo MRI safety training with MRSO.
Radiology Review	• MRI images are reviewed weekly with a neuroradiologist for incidental findings.

### Incidental findings. Safety Events, and statistical analysis

Consecutive patients from March 1, 2020, to May 31, 2022, who were scheduled for MRI sim after having completed CT simulation for head and neck radiotherapy were included for analysis. Patients were selected for MRI sim on the basis of patient and disease factors listed in Table 2. The purpose of this study was to retrospectively review patients scheduled for both CT and MRI sims to report the incidental findings or safety events observed from MR sim. Incidental findings were identified during weekly quality assurance rounds as a joint enterprise of head and neck radiation oncology and neuroradiology [17]. Incidental findings were categorized as local recurrence or progression, distant metastasis, or non-oncologic, and the proportions of each category were compared descriptively between CT and MRI sim.

**Table 2:** Common Reasons for MRI Sim over CT Sim Alone.

Patient Factors	Disease Factors
• Dental amalgam Iodine contrast contraindication Complex surgical reconstruction	• Skull base involvement Intracranial extension Perineural tumor spread Ill-defined soft tissue invasion by CT (e.g. extranodal extension)

Safety events were instances where scheduled MRI sims were not completed due to the MRI technologist identifying MRI-incompatible devices or objects at the time of sim. Safety events were compared descriptively to other reasons for not completing the scheduled MRI (claustrophobia, technical, MD decision, and staffing). Adverse events from MRI sim were also reviewed for any observed thermal, mechanical, projectile, or acoustic-related injuries. Categorical variables between completed and not completed MRI sims were compared using the Chi-Square test and continuous variables were compared using the Mann-Whitney *U* test with a p-value of < 0.05 considered to be statistically significant. This retrospective study was considered of minimal risk to patients and was exempt from Institutional Review Board (IRB) review.

## Results

### Patient and tumor characteristics

148 of 169 MRI sims (88 %) were completed as scheduled and 21 (12 %) were not completed (Table 3). Patients who did not complete MRI sim were more likely to be treated for non-squamous head and neck primary tumor (p = 0.016) and were being treated post-operatively (p < 0.001). There was also numerically a greater proportion of patients unable to undergo MRI sims who had gastrostomy feeding tubes though not significant (p = 0.072).

**Table 3:** Patient and tumor characteristics.

	Completed MRI-sim (n = 148)	Did Not Complete MRI-sim (n = 21)	p
Age	65 (54–72)	63 (50–68)	0.71
Male Sex	99 (67 %)	17 (81 %)	0.19
Required Interpreter	24 (16 %)	4 (19 %)	0.74
Gastrostomy Tube at Sim	25 (17 %)	7 (33 %)	0.072
Squamous Histology	114 (77 %)	11 (52 %)	**0.016**
Post-Operative	50 (34 %)	15 (71 %)	**< 0.001**
Duration of MRI (min)	32 (28–37)	n/a	

### MRI sim incidental findings

**Fig. 2:**
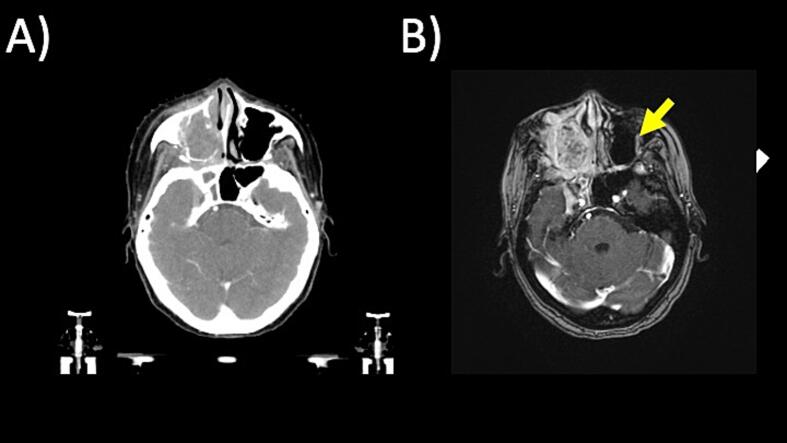
A patient with a right maxillary sinus adenoid cystic carcinoma noted on MRI sim to have unsuspected contralateral perineural tumor spread of the left infraorbital nerve. (A) CT and (B) MRI sim images are shown.

**Fig. 3:**
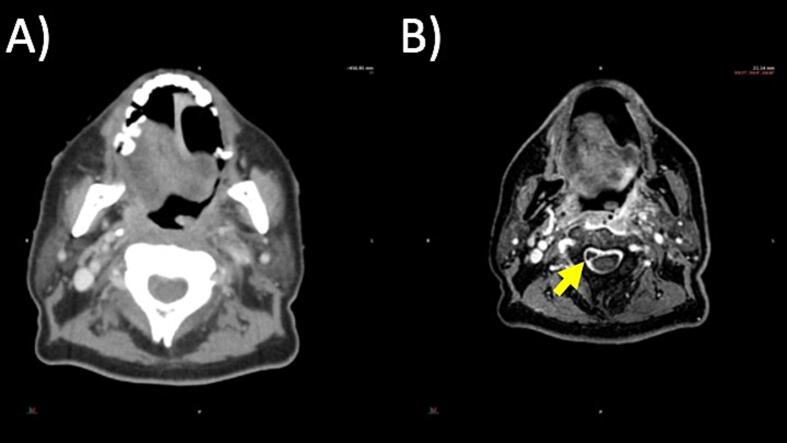
A patient with oropharyngeal squamous cell carcinoma recently treated with transoral resection was noted on MRI sim to have a phlegmon and epidural abscess at the C2-C3 level from iatrogenic diskitis-osteomyelitis. (A) CT and (B) MRI sim images are shown.

CT and MRI sim scans each had 17 incidental findings (Table 4). There were 14 post-operative bed recurrences detected by both CT and MRI. There was only one case of local disease progression by MRI and not CT in a case of right maxillary sinus adenoid cystic carcinoma with contralateral infraorbital perineural tumor spread detected by MRI sim images (Fig. 2). CT simulation detected 3 cases of new metastases in lungs, which were outside the scan parameters of MRI sim. MRI sim detected one case of dural venous thrombosis and one case of cervical spine epidural abscess, which were not detected by CT simulation (Fig. 3).

**Table 4:** Comparison of CT and MR Incidental Findings in 148 Patients Undergoing both CT and MR Simulations.

Incidental Finding	CT (n = 17)	MRI (n = 17)	Comments
Local Recurrence or Progression	14 (9 %)	15 (10 %)	One case of MRI-detected perineural tumor spread, the remaining are post-operative bed recurrences
Distant Metastasis	3 (2 %)	0	Three cases of new lung metastases
Non-Oncologic	0	2 (1 %)	Dural venous thrombosis, epidural abscess

### MRI sim safety events

Among the 21 aborted MRI sims, the most common reason was due to safety events flagged by the MRI technologist (n = 8, 38 %) because of the presence of metal or a medical device that was not noted at the time of initial screening by the administrative coordinator (Table 5). These 8 events consisted of: remote injury leaving metal near the eyes, history of exposure to bomb shrapnel, or presence of cardiac device not MRI conditional at 3 T, orthopedic hardware recommended for 1.5 T MRI, and reconstruction hardware (orbital mesh and eyelid weight) with inadequate manufacture and model data at the time of MRI sim. Aside from safety events, other reasons for not completing MRI sim studies included claustrophobia (n = 5, 24 %), MRI machine maintenance (n = 4, 19 %), MD deciding patient was unfit for MRI simulation (n = 2, 10 %), and unexpected unavailability of the MRI technologist (n = 2, 10 %). There were no documented adverse events, namely no thermal, mechanical, projectile, or acoustic injuries in patients who completed MRI sim.

**Table 5:** Safety and Other Events Leading to Aborting MRI Simulations.

Event Type	Count (n = 21)	Comments
Safety	8 (38 %)	Neurostimulator (1), neurovascular embolization coils (1), orthopedic hardware (1), cardiac device (1), injury (2), hardware from surgical reconstruction (2)
Claustrophobia	5 (24 %)	Refractory to anxiolytics
Technical	4 (19 %)	MRI needed maintenance
MD Decision	2 (10 %)	One post-laryngectomy patient could not remain supine and required a slant board for CT sim, which could not be used for MRI sim. One elderly patient with soft tissue reconstruction in the mouth and tracheostomy had difficulty completing CT sim due to salivary secretions and MRI was not felt to be possible by MD.
Staffing	2 (10 %)	MRI tech was unexpectedly unavailable

## Discussion

In recent years, interest in the use of MRI in radiotherapy has increased considerably. Compared to CT, MRI offers superior soft tissue contrast for more accurate target delineation. The incorporation of MRI to CT-based planning improves target definition for many head and neck subsites [18], [19], [20], [21]. Furthermore, repeating MRI simulations during head and neck radiotherapy may allow for early response assessment to help guide treatment adaptation [22]. The development of linear accelerators with integrated MRI scanners (MR-Linac) and synthetic CT generation methodologies from MRI sim images has made MR-only radiotherapy possible [23], [12], [13]. As a result, there will be an increasing number of MRI scanners in radiation oncology departments and consequently, an increasing number of professionals involved with radiotherapy will be confronted with the challenges posed by MRI technology. This study retrospectively reviews the incidental findings and safety events that were observed at a single institution during introduction of MRI sim for head and neck radiotherapy planning.

Since radiation oncologists are generally more familiar with CT than MRI, incidental findings from MRI sim may be more likely to be unrecognized without diagnostic radiology support. Retrospective studies have reported 15–20 % of CT sims have incidental oncologic and non-oncologic findings, most of which are not clinically significant [24], [25]. In this study, among patients who underwent both CT and MRI sim, incidental findings were observed in similar proportions from CT or MRI sim (11 %). However, MRI sim detected non-oncologic important incidental findings in two patients that were not observed on the corresponding CT sim. MRI sim revealed an incidental findings of a dural venous thrombosis and an epidural abscess requiring further clinical management, both of which were identified by a neuroradiologist at the time of contour delineation review [17].

The hazards of a MRI environment are often underestimated [26]. A 10-year review of 1548 MRI-related adverse events reported to the US Food and Drug Administration found that the most common hazards were thermal (59 %), mechanical (11 %), projectile (9 %), and acoustic (6 %) [27]. MRI scanners in radiation oncology can be particularly hazardous as most personnel may not be familiar with the risks or required screening procedures [14]. Furthermore, head and neck radiotherapy patients often need customized immobilization devices [28] or have undergone complex surgical reconstruction with implanted devices, all of which need to be ensured to be MRI-safe. Overall, there were no adverse events attributed to MRI sim studies in this study, which is largely due to having an MRI technologist with credentialing in MRI safety. The MRI technologist cancelled 8 MRI sims in patients who passed the initial MRI screen after identifying metal or devices that were deemed unsafe or with inadequate data on safety to proceed. Even though safety events were the most common reason (38 %) for aborted MRI sims, they were relatively rare in the entire cohort (5 %) and 88 % of patients completed MRI sims as planned.

The main limitations of this study are its retrospective nature, small sample size, and low number of outcomes of interest. We plan further prospective investigations MRI sim for head and neck radiotherapy to further evaluate incidental findings and near-miss events.

Overall, this study suggests that radiation oncology departments with dedicated MRI simulation scanners would benefit from diagnostic radiology review for incidental findings and having therapists with MRI safety credentialing to catch near-miss events.

## Funding

This work was completed without external grant assistance.

## Waiver of patient consent

This is a retrospective case study. Patient consent has been waived by Ethic committee.

## Declaration of competing interest

The authors declare the following financial interests/personal relationships which may be considered as potential competing interests: The authors declare that the research was conducted in the absence of any commercial or financial relationships that could be construed as a potential conflict of interest.
